# A preliminary study on home range and daily travel distance of François’ langur in a high-altitude area

**DOI:** 10.1007/s10329-022-01041-w

**Published:** 2022-12-29

**Authors:** Huaiqing Deng, Wenqing Hong, Jiang Zhou, Jixiang Li

**Affiliations:** 1 School of Life Sciences, Guizhou Normal University, Guiyang, 550001 China; 2 State Engineering Technology Institute for Karst Desertification Control, School of Karst Science, Guizhou Normal University, Guiyang, 550001 China; 3 Guizhou Kuankuoshui National Nature Reserve Administration, Suiyang, 563300 Guizhou China; 4 Graduate School, Guizhou Normal University, Guiyang, 550001 China

**Keywords:** Primates, Home range, Daily travel distance, Kuankuoshui, Karst

## Abstract

François’ langur (*Trachypithecus francoisi*) is an indicator species living in Karst rocky habitats. To understand the François’ langurs ecological adaptation to high-altitude habitat, we used the field tracking method to collect home-range data and daily travel distance of François’ langur at high-altitude (800–1400 m) areas of Kuankuoshui National Nature Reserve between April 2008 and March 2009. The results revealed the following: (1) according to the grid cell method, the home range of François’ langurs was estimated to be 50.7 ha. The area in the winter-spring season was larger than in the summer-autumn season (37.7 vs. 27.7 ha). According to the minimum convex polygon method by GIS, the home range of François’ langurs in the winter-spring season and the summer-autumn season was 123.5 and 68.8 ha, respectively. The whole-year home range of François’ was clearly larger than the grid cell method (140.4 vs. 50.7 ha). (2) The home range of François’ langurs had clear superposition. Langur's core areas were concentrated in three small areas, which only occupied 18.2% of the home range. (3) The langur had a short daily travel distance (230–1115 m) and significant seasonal differences. The summer-autumn season moving distances being obviously shorter than the winter-spring season (517 vs. 785 m). (4) With the decrease in the availability of food resource in winter-spring season, the home range and daily travel distance of François’ langurs significantly increased. (5) Living in the high altitudes, François' langurs tended to form a larger group (≥ 8 individuals), which is larger than other groups at lower altitude, had the larger home range, and had shorter daily travel distance. Our results indicate that colobines’ survival strategies tend to form a larger home range and shorter day-traveling distance to adapt to high-altitude and low-temperature habitat.

## Introduction

*Trachypithecus francoisi*, which belongs to the *Trachypithecus* genus, colobines, is a unique primate that only survives in karst area of Asia and a first-class protected wild animal in China (Groves 2001). It occupies an important position in the protection of tropical-subtropical forest ecosystems from China to Southeast Asia. Historically, the species was widespread in the northern provinces of Vietnam and parts of southwestern China; however, habitat destruction and poaching have resulted in fragmentation and a dramatic decrease in the population (Groves 2001). According to relevant literature, there are no more than 200 individuals left in Vietnam (Nadler and Brockman 2014), while some 1700 individuals are distributed in Guangxi, Guizhou, and the Chongqing areas of China. A total of 554 individuals of 72 groups were reported in Mayanghe Nature Reserve (Niu et al. 2016), 195 individuals of 29 groups in Kuankuoshui Nature Reserve (Yao et al. 2016), 119 individuals of 16 groups in Yezhong Nature Reserve (Deng and Zhou 2018), and 120–146 individuals of 18 groups in Dashahe Nature Reserve, Guizhou Province (Huang 2008).

A home range is an area occupied by a wild animal over a long period of time, where the animal engages in its daily activities, including foraging, reproduction, hiding, and child-rearing (Burt 1943). Home-range studies further the understanding of the complexity of animal behavior and ecology, including foraging strategies, physiological and morphological specializations, and social relationships. The ecological research of François' langurs is mainly carried out in Mayanghe National Nature Reserve in Guizhou. A previous study has found that the seasonal behavioral changes of François’ langur in appropriate habitats (suitable terrain conditions and vegetation coverage, less human disturbance) were mainly affected by habitat temperature and distribution of food resources in Mayanghe National Nature Reserve, with obvious regularity and active behavior that mainly occurred in the afternoon; however, there were no significant changes in inappropriate habitats (Luo 2002). Another study suggested that the home range of François’ langur in Mayanghe National Nature Reserve was influenced by edible plant species and human factors; its shape became narrow and long, and its home range in spring and autumn was larger than that in summer and winter. In addition, there was overlap in the spatial location of groups' home ranges, but not temporal overlap in the use of this space (Chen et al. 2001). The existing research is qualitative description. There is a lack of quantitative data in Guizhou, which is not convenient to follow-up studies. Zhou studied the feeding habits, activity rhythm, home range, habitat selection, and roosting site of François’ langur in Nonggang Nature Reserve, Guangxi, and discussed the adaptability of François’ langur to typical Karst Rocky Mountains (Zhou 2005).

Primates can adjust their home ranges and daily travel distances based on the availability of major foods. The whole-year biggest home range of François’ langur in Guangxi was 69.3 ha (Table 1). The home range in the dry season was significantly larger than that in the rainy season (Huang et al. 2011), and the core area (52%) occurred within a small area (only 22% of their home range) (Zhou et al. 2007). The average daily travel distance of François’ langur living in Nonggang Nature Reserve was 541 m (Zhou 2005), and that of François’ langur in Fusui County was about 438 m, with significant seasonal changes. The daily travel distance in the dry season (471 m) was significantly larger than that in the rainy season (403 m) in Nonggang Nature Reserve (Table 1) (Huang et al. 2011).

**Table 1:** Comparison of group size, altitude, home-range size, and roaming distance of Francois' langurs in different nature reserves

Study sites (nature reserve)	Group size	Population density (number /km^2^)	Altitude (*m*)	Home range (ha)	Daily travel distances (*m*)	References
Nonggang	9	–	300–650	28.7	661	Huang et al. (2011)
Fusui	4	6.0	400–600	19.0	438	Zhou et al. (2007)
Nonggang	12	1.4	300–650	69.3	541	Zhou (2005)
Kuaikuoshui	12	4.0	800–1400	50.7–140.4	462	This study

Previous studies on the ecology of François’ langur mainly focused on low-altitude areas in Guangxi, which are generally below 600 m. The distribution area of François’ langur in Guizhou Province was generally at a high altitude, with an elevation range between 300 and 1400 m in Mayanghe Nature Reserve, while that of Kuankuoshui Nature Reserve was at 650–1760 m, and that of Dashahe and Yezhong Nature Reserves was at 560–1939 m and 780–1680 m, respectively. No in-depth studies or systematic research has been carried out on François’ langur in the high-altitude areas of Guizhou Province. François’ langur in Guangxi mainly lives in typical Karst Rocky areas. The main composition of the rock is carbonate, and the slope of the mountain is not high in Guangxi, which is different from the habitat of the François’ langur in Guizhou.

**Fig. 1:**
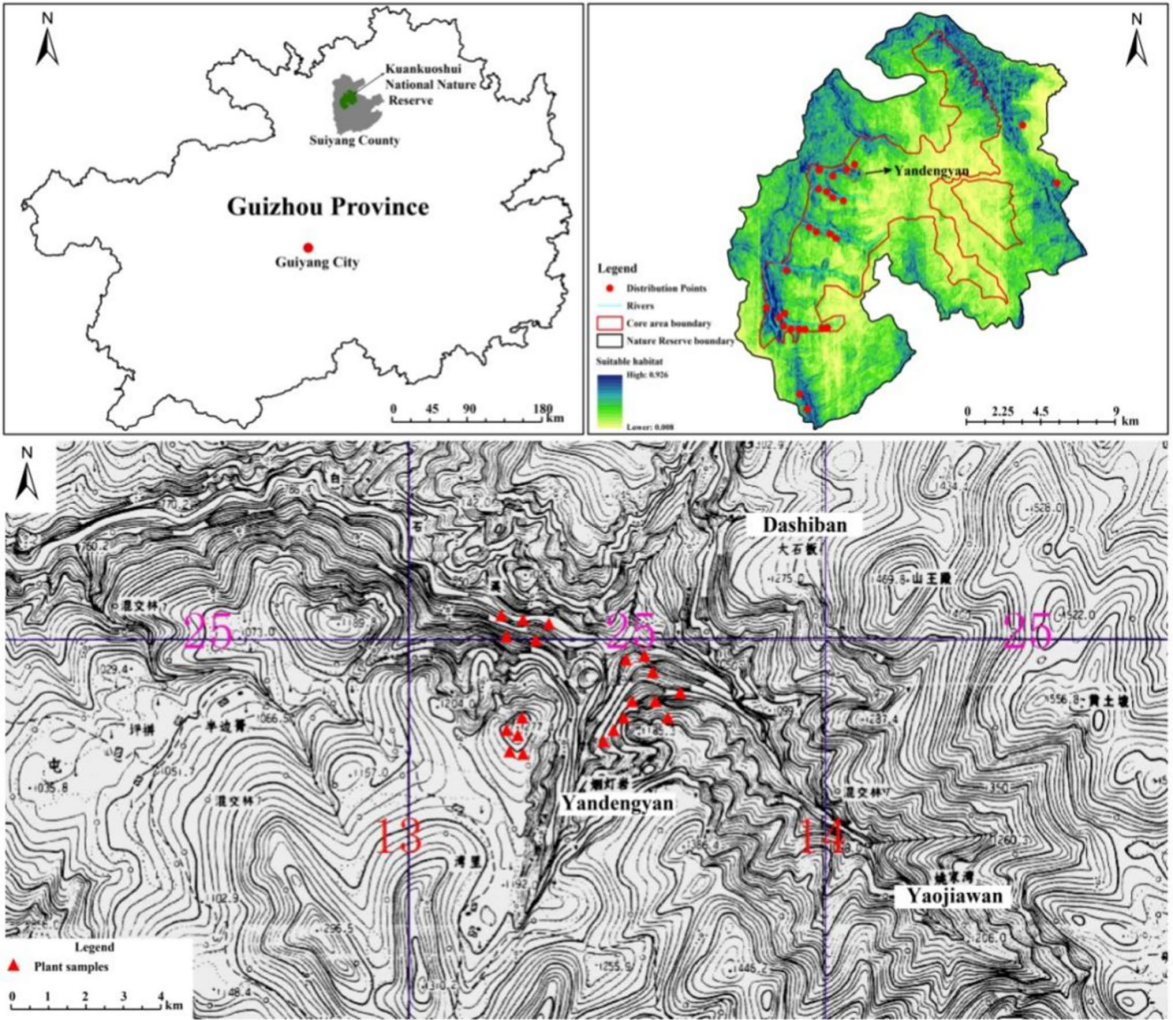
Map of the target monkey group activity areas and plant samples

In this study, a group of François’ langurs in Yandengyan, a high-altitude area in the Kuankuoshui Nature Reserve, were selected as the research subjects (Fig. 1), whose home range and daily travel distance were tracked for 1 year. Combined with the plant phenology survey of the François’ langur’s active area, this study explored the area size of the François’ langur’s home range in different seasons, the potential overlapping in the home range, the daily travel distance, and its relationship with the plant availability in the high-altitude area. These data were then compared with François’ langurs distributed in the low-altitude carbonate karst region in Guangxi to analyze any differences between the two. Also, the influence of the altitude difference on the home range and daily travel distance of François’ langur was discussed to understand François’ langurs’ adaptability to high-altitude habitat as a survival strategy.

## Study animal and method

### Study sites and subjects

Kuankuoshui Nature Reserve is located on the eastern slope of the Daloushan Mountain Range in the north of Guizhou, and covers an area of 26,231 ha. The middle part of its terrain is high, and the surrounding is low. The terrain is distinctly cut, and the relative height difference is large, with the altitude ranging between 650 and 1762 m. Mountain rocks are dolomitic limestone-bicarbonate or potassium aluminate, a non-calcium carbonate karst landform (Yu et al. 2004). The research site was located in the Yandenyan area of the reserve, which is located in the subtropical humid monsoon climate zone with an average annual temperature of 14.4 °C. The high temperature is concentrated from April to October, and the daily average temperature was ≥ 14.5 °C. The low temperature is from November to March of next year with ≤ 10.0 °C. The lowest temperature is in the month of January, with an average temperature 0.19 °C (Liu 2009). Total rainfall is 1324 mm, mainly concentrated in April–October, which accounts for 79.6% of the annual rainfall. According to the average temperature and rainfall, the year can be divided into two seasons: summer-autumn season from April to September of the current year, and winter-spring season from October of the current year to March of the following year. In the following description, Jan-Mar, Apr-Jun, Jul-Sep, Oct-Dec = quarters, and the winter-spring and summer-autumn = seasons.

The target monkey group initially had 10–11 individuals in 2007, which included eight adult and 2–3 infants, but later the infants disappeared from the group. Then two infants were born in December 2008, and two infants were born in January and February of 2009. So, the target monkey group consisted of 12 monkeys (one adult male, seven adult females; four newborns) at the later stage of the study. Their activity area was Yandengyan in E 107°05′25″-107°6′21″, N 28°13′52″-28°14′17″, the opposite mountain slope and the single mountain between these, as well as both sides of the canyon west of Yaojiawan and around the Dashiban (Fig. 1). Yandengyan, where this monkey group lived, was only about 300 m away from a farmer’s residential area, and this monkey group had adapted to the presence of observers, which made it relatively easy to follow and observe them. The area was a dolomitic karst peak-cluster canyon, with the peak on both sides reaching > 1400 m. The altitude at the bottom of the valley was 700–800 m, and the relative height difference was > 600 m. The area was dominated by evergreen broad-leaved forest.

## Methods

### Vegetation and phenology survey

In order to understand the vegetation conditions of the habitat, 20 quadrats were set up at the bottom of the valley, the middle and upper part of the mountain, and the top of the peak of the study area. Five of the quadrats were located at the bottom of the valley or flat land, at an altitude of 750–900 m; eight of the quadrats were located on the slopes of different altitudes in the middle of the mountain; seven of the quadrats were located on the top of the mountain at altitudes of 1200–1400 m (Fig. 1). Vegetation type classification and quadrat setting referred to the vegetation in Guizhou (Huang 1988). The quadrat size was 10 × 10 m, which was determined according to the plant distribution and the terrain conditions of the study area. After each quadrat was set, it was circled with quadrat rope in the field, and all trees with diameter at breast height (DBH) ≥ 5 cm and plant height ≥ 1.5 m were marked with a marker pen, and the height and crown diameters of each marked tree were recorded. The phenological changes of all marked trees in the quadrat were regularly monitored in the middle of each month, and the growth of their young leaves, flowers, and fruits were recorded. Finally, the monthly tree index was calculated. The calculation method was the following: Tree index = (Ni/N) * 100%, where Ni is the number of trees with young leaves, flowers, or fruits and N is the total number of trees in the quadrat. Although this method cannot truly reflect plant production, it can provide an estimate. Besides, this approach has been widely used in primate research (Britt et al. 2002; Di Fiore 2003). The Mann–Whitney *U* test was used to examine the available differences of leaves, flowers, and fruits in different seasons.

### Sampling of home range and daily travel distance

The field tracking method was used to observe the home range and daily travel distance of the target François’ langur group between April 2008 and March 2009. The fieldwork was performed 15–20 days per month, while the phenology monitoring lasted 3 days, and the fieldwork was 216 days in total. However, due to the limitations of field terrain conditions, observation subjects were often hidden or escaped and could not be continuously tracked and observed, so it was difficult to achieve continuous sampling throughout the day.

Before starting field tracking observation, transparent paper with grids was superimposed on a 1:10,000 map, where the size of the grid was 0.5 × 0.5 cm so that each grid represented a quadrat of 50 × 50 m. At the same time, horizontal and vertical coordinate axes were set on the grid map to record the position of each grid. The grid size of 50 × 50 m was chosen because the distance between the monkey groups and individuals was found to be within 50 m for most of the time when observed in the field.

During observation, the monkeys were tracked with binoculars (Sportstar EX 10*25). When collecting data, the GPS points of the monkeys were recorded every 30 min from the first time when the monkeys were seen. The general follow observation distance between 100 and 300 m. Then the corresponding positions of the monkeys were marked on the 1:10,000 map. Several observation points were set up in Yandengyan, Lanyapo, Dashiban, Yangmeiyakou, and Longdongwan, where the monkeys were most active. For the places which could not be directly reached by the observers, the monkeys were positioned by estimating the distance and orientation of the monkey group from the observation point. If the roosting site of the monkey group on the previous day could be determined, sampling started when the monkey group was active on the next day, and the observation was continued until the monkey group entered the roosting site if conditions permitted. Due to the heavy fog in January, it was difficult to track and observe the monkeys, and the data were limited. Over the study period, a total of 459 h of locations data in 102 days were collected (App. 1).

### Data analysis

With the help of a digital topographic map of the Kuankuoshui Reserve, combined with GPS sites recorded in the field, the grid method and minimum convex polygon (MCP) method were used to estimate the home-range size of the monkey group throughout the year and during each season. The daily travel distance sites of the monkey group were recorded once every half an hour. Because of the terrain conditions, it is very difficult to be able to monitor the activities sites all day, so the daily travel distance analyses were limited to days on which the group's location was record at least ten times. The daily travel distance was represented as the sum of the straight-line distance between every two consecutive recording locations in same day.

In order to analyze the intensity of use by the monkey group in their home range, the proportion of use for each 50 × 50 m grid (the ratio of the number of site records of each grid to the total number of site records) in each season and for the full year was calculated. Also, the area of the main active area of the home range of the monkeys in each season and throughout the year was estimated. The core area referred to grids where the sum of use intensity accounted for 60% of the total site records (Zhou 2005). We calculated the home-range overlap between adjacent months and seasons. The degree of overlap was estimated with the following formula: overlapping area/ adjacent month or season home range on average multiplied by 100%. The Mann–Whitney *U* test was used to examine seasonal differences in home range use and daily travel distance. The Spearman's rank correlation tests were used to analyze relationships between moving distance and food availability. Data analyses, examination, and comparison were performed on Microsoft Excel and SPSS 17.0 statistical software, significant standard set to *P* < 0.05.

## Results

### Vegetation phenology changes

**Fig. 2:**
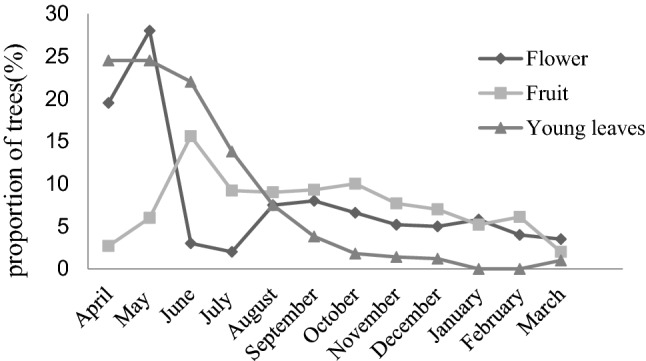
Monthly availability of young leaves, fruits, and flowers based on the data of 20 quadrates in Kuankuoshui Nature Reserve from April 2008 to March 2009

There were 417 plant trees of 52 species marked in the research area; the most common species were Ericaceae, Fagaceae, and Theaceae. The valley bottom and the middle and lower parts of the mountain in the studied area were subtropical evergreen broad-leaf forest and evergreen deciduous broad-leaved mixed forest formed by *Bothrocaryum controvers*, *Lindera communis*, and *Dendrobenthamia hongkongensis.* The middle and upper parts of the mountain and the top were mainly coniferous forests and coniferous broad-leaved mixed forests formed by *Fokienia hodginsii*, *Cunninghamia lanceolata,* and Fagaceae plants. There were obvious seasonal phenological changes in vegetation. The monthly availability of young leaves, flowers, and fruits are shown in Fig. 2 and App. 2. The number of young leaves, flowers, and fruits that François’ langurs preferred to eat in the high-altitude area of the Kuankuoshui Reserve showed obvious seasonal variation. The young leaves were most abundantly available from April until June, and the flowers and fruits peaked in May and June. In the summer-autumn season, young leaves and fruits were significantly more available than that in winter-spring season (young leaves: *Z* =  – 2.725, *P* < 0.01; fruit: *Z* =  – 2.119, *P* < 0.05). There was no significant seasonal difference in flower availability (flower: *Z* =  – 0.303, *P* > 0.05), while the peak occurred in June.

## Home range

### Home-range size

During the study period, a total of 102 days of records were obtained, and 20–150 sites were obtained per month. A total of 918 sites of the monkey group were recorded (**App.** 1). As January was foggy and difficult to observe, the number of obtained samples in this time was few.

**Fig. 3:**
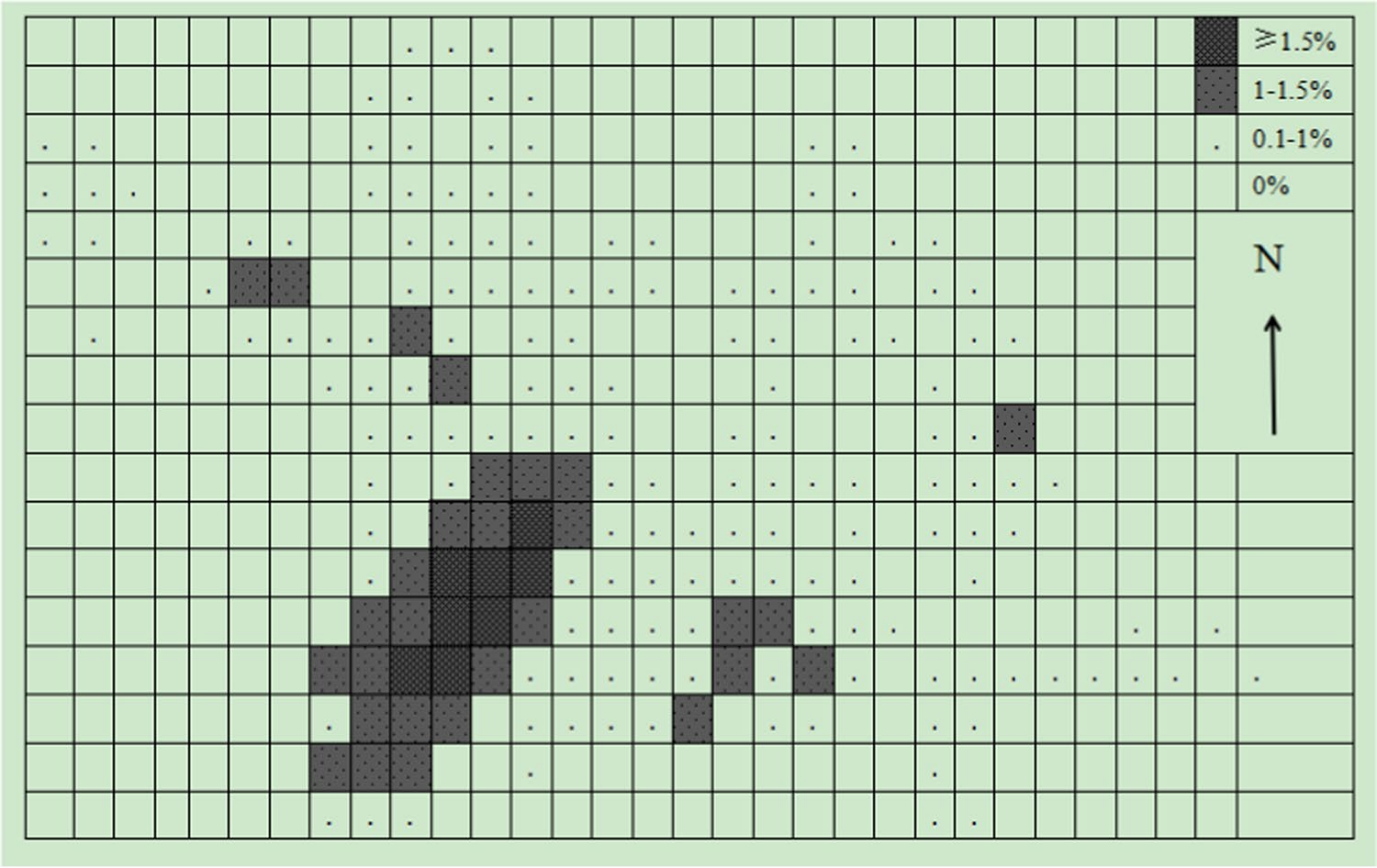
Use percentage of different 50 × 50-m quadrat in the whole-year home range

**Fig. 4:**
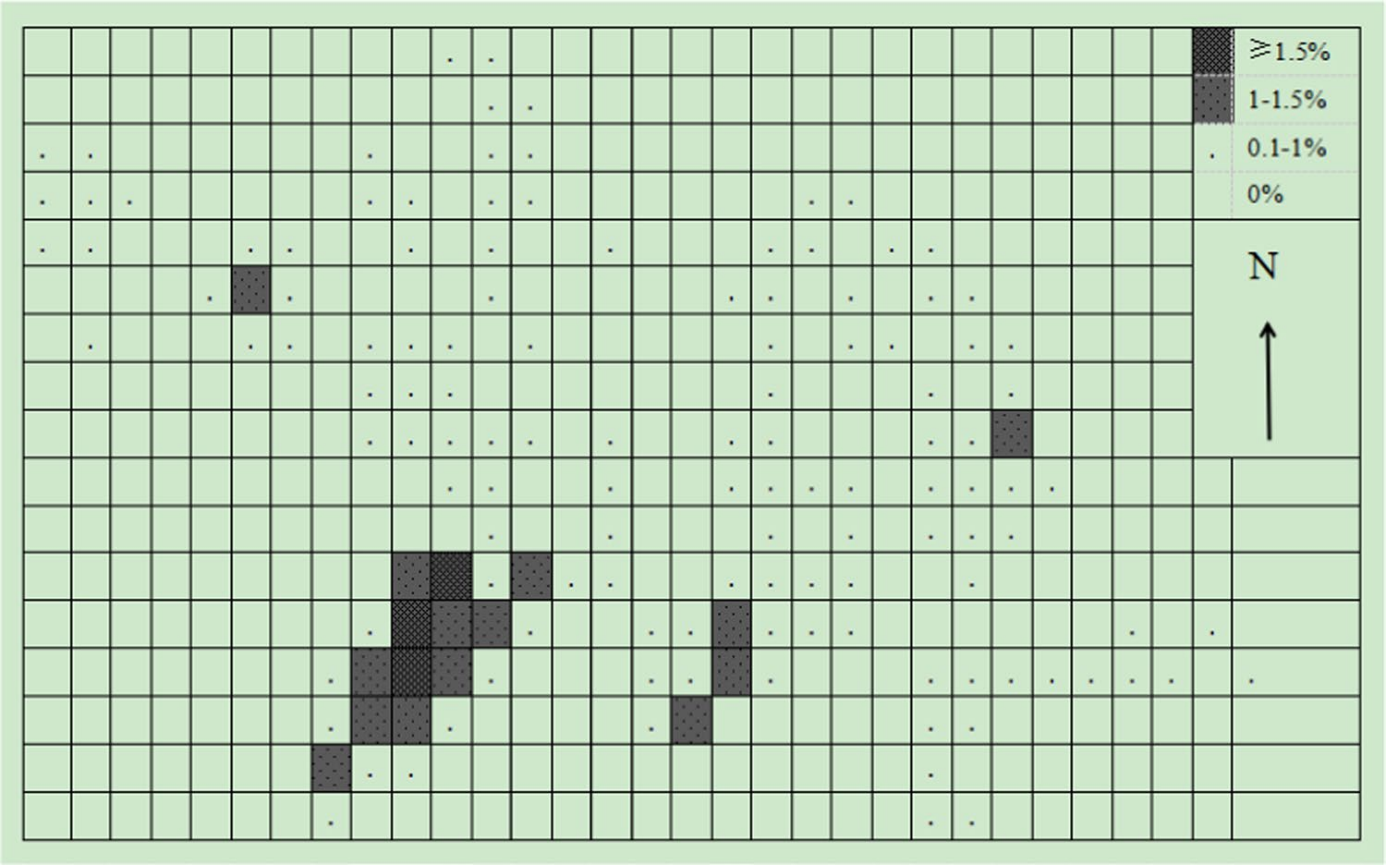
Use percentages of different 50 × 50-m quadrats in winter-spring season home range

**Fig. 5:**
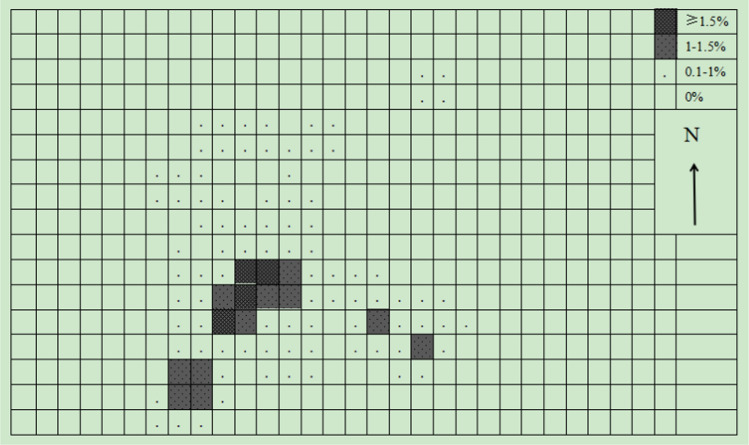
Use percentages of different 50 × 50-m quadrats in summer-autumn season home range

The grids method results show that there were 193 grids of 50 × 50 m used by the monkeys in a year. Another ten grids were clearly within the monkey’s range of activity and were also calculated within the home range, although they were not directly observed to be used by monkeys. Therefore, the home range of the target monkey group included 203 grids of 50 × 50 m, which was 50.7 ha (Fig. 3). There was a significant seasonal difference in the home range of monkey groups (*Z* =  – 0.194, *P* > 0.01). The home range in winter-spring season was larger than that summer-autumn season. In the winter-spring season, 148 grids of 50 m × 50 m were utilized by the monkeys, and 3 unused grids were obviously within the activity range of the monkeys. Therefore, the home range of the monkeys in the winter-spring season was 37.7 ha (Fig. 4). On the other hand, in the summer-autumn season, the monkeys actually used 105 grids of 50 × 50 m, and there were another six unused grids that were obviously within the activity range of the monkey. Therefore, the home range of the monkeys in the summer-autumn season was 27.7 ha (Fig. 5).

**Fig. 6:**
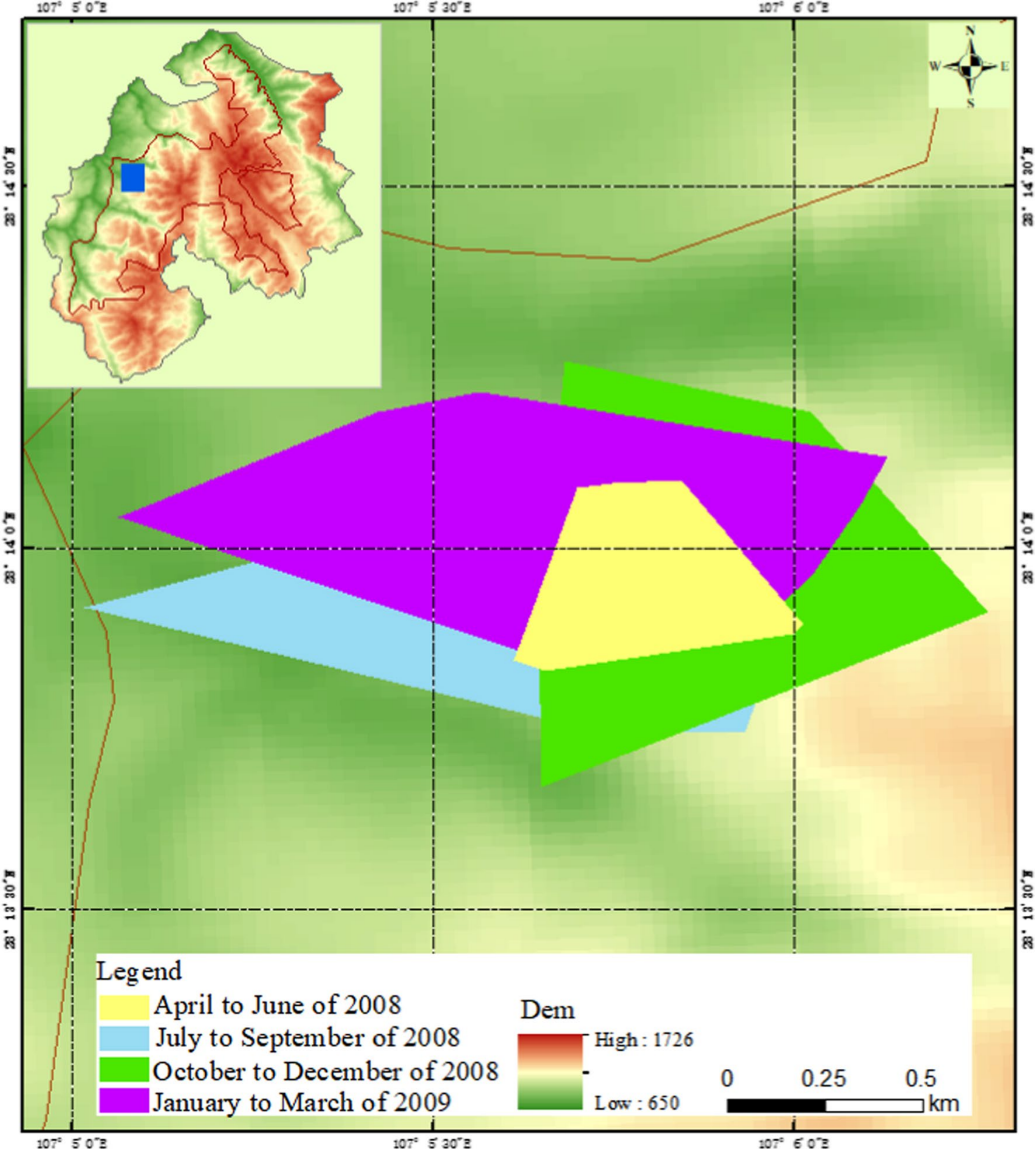
The François’ langurs home range in each quarters calculated using a minimum convex polygon (MCP) method

**Fig. 7:**
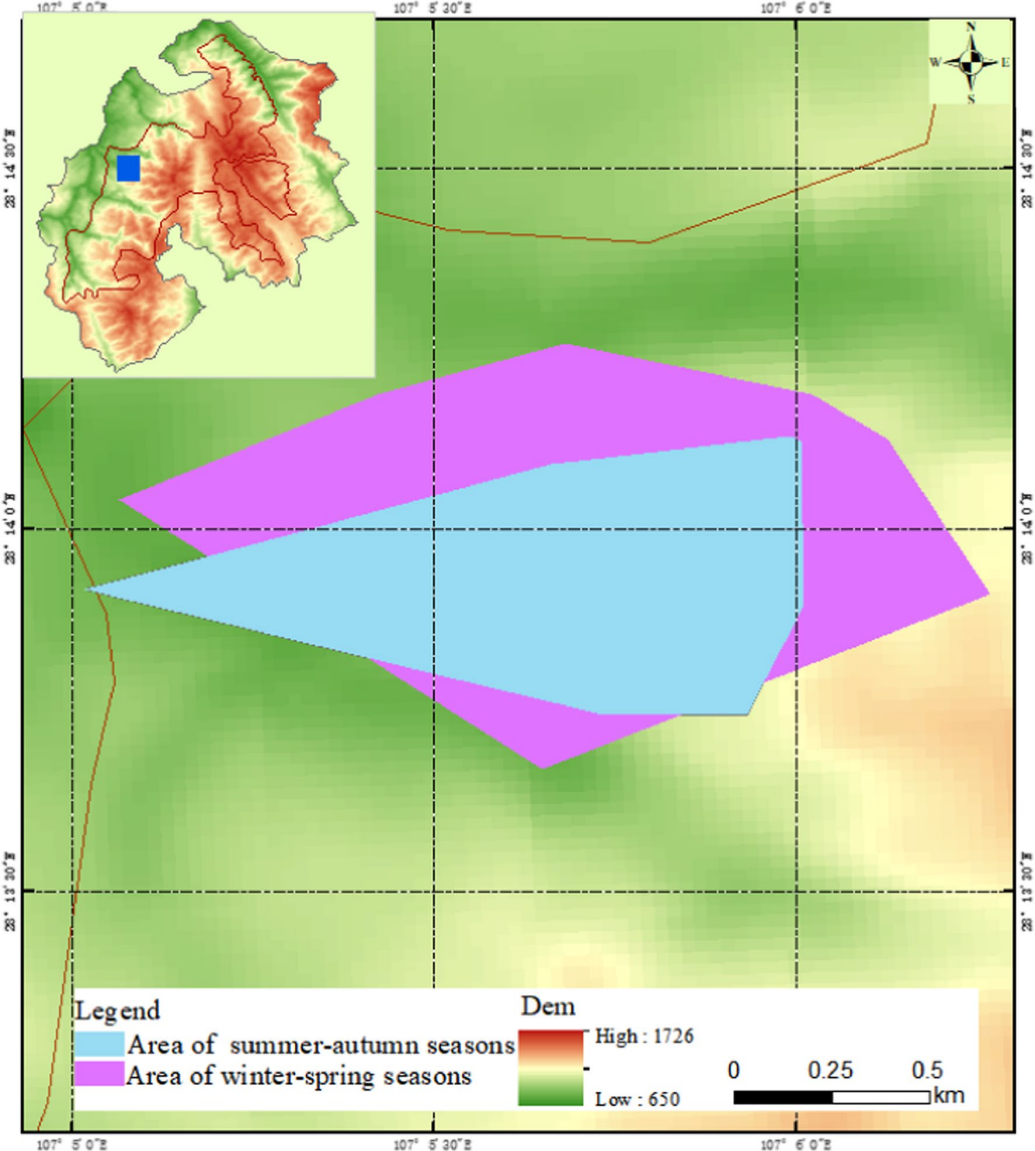
The François’ langurs two seasons home range in Kuankuoshui Nature Reserve calculated using a minimum convex polygon (MCP) method

**Fig. 8:**
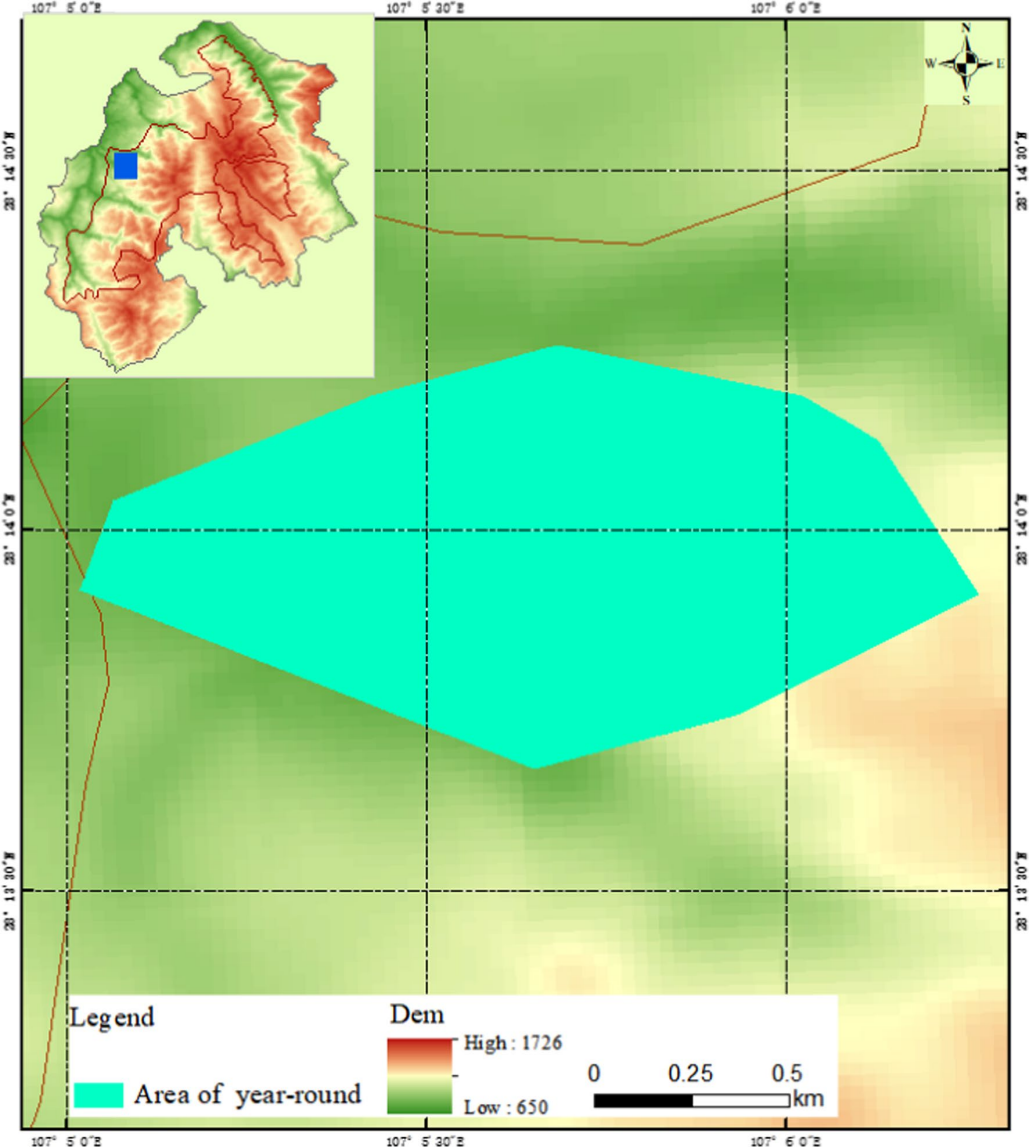
The François’ langurs whole-year home range in Kuankuoshui Nature Reserve calculated using a minimum convex polygon (MCP) method

The seasonal home range of François’ langurs calculated by the GIS system using the MCP method was 20.8, 67.1, 66.9, and 71.1 ha, respectively, from the second quarter of 2008 to the first quarter of 2009 (Fig. 6). The home range was 68.8 and 123.5 ha, respectively, in summer-autumn season and winter-spring season (Fig. 7). The whole-year home range was 140.4 ha (Fig. 8), which was significantly larger than that calculated by the grid method. That was because some of the areas where no François’ langur was active were also being counted, which might produce home range estimates larger than they actually were.

### Overlap in core area use within the home range

The François’ langurs did not evenly use their home range. Among 453 site records, 58% of site records came from 37 grids of 50 × 50 m, accounting for only 18.2% of the home range (Fig. 3). The core areas of the monkeys were mainly concentrated in three activity centers, which were located near the Yandengyan platform, in the northeast of Lanyapo (the western slope of Yandengyan) and in the south of Longdongwan. In all regions, there were 29 grids (7.25 ha), and the using frequency of each grid accounted for more than 1.0% of the total used grids, accounting for only 18.2% of the home range. Among them, there were eight girds in the Yandengyan area, and the use frequency of each grid accounted for more than 1.5% of the total of used girds.

François' langurs will reuse the same place at a different time in home range. The mean (SD) overlap degree of the monkey group’s home range in each season was 25.4 ± 1.95%. The highest overlap degree was 27.7% between spring and summer, the minimum was 21.6% between summer and autumn. The largest overlap degree between months was in April and June (37.9%), and the smallest was in February and March (11.7%). Although the months overlap degree of home range in the summer-autumn season was greater than that in the winter-spring season, the difference was not significant (Mann–Whitney *U* test *Z* =  – 0.527, *P* > 0.05). The total overlap area of home range between summer-autumn season and winter-spring season was 12.5 ha, accounting for 45.1% of home range in summer-autumn season and 33.1% of home range in winter-spring season, respectively.

### Encounter behavior between groups

There were two groups of François’ langurs living in the study area, but the non-target monkey groups hardly entered the home range of the target monkey group. During the study period, the non-target monkey group entered the home range of the study monkey group only once. First, they confronted on the north side of Lanyapo, which was about 300 m away from the target group on the top of Yandengyan, threatening each other with loud calls. Later, the non-target group fed in the valley floor on the north side and moved west along the Baishixi ditch after about 20 min. After more than half an hour, the target group moved down from the top of the mountain to the Yandengyan platform; there was no direct physical contact or injuries between the monkey groups.

## Daily travel distance

**Fig. 9:**
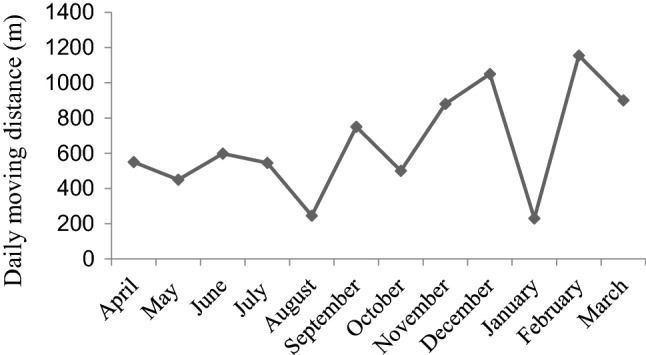
The François’ langurs largest daily travel distance in each month from April 2008 to March 2009

During the study period, the average daily travel distance of the monkey group was 462 ± 232 m; the shortest was 230 m in January 2009, and the longest was 1155 m in February 2009 (Fig. 9). There was a significant seasonal difference in daily travel distance (*Z* =  – 0.173, *P* < 0.01). The availability of leaves(*r* =  – 0.38, *P* > 0.05), flowers (*r* =  – 0.43, *P* > 0.05), fruit (*r* =  – 0.16, *P* > 0.05) and moving distance are inversely correlated, but there is no significant difference. With the decrease in the availability of young leaves, flowers, and fruits in winter-spring season, the daily travel distance of François’ langurs substantially increased. The average monthly maximum daily travel distance in the summer-autumn season (517 m) was obviously smaller than that in the winter-spring season (785 m) (*Z* =  – 2.373, *P* < 0.05).

## Discussion

### Relationship between home range and population density and group size of François’ langurs

We found a larger home-range size at our study site. The difference in population density might be one factor causing the difference in home range size. A previous study reported a significant negative correlation between home range and population density of East African colobus monkey (Fashing 2001). When the population density is low, primates can expand their home range according to the population size. When population density is high, the home range is reduced by competition from neighboring monkey groups. The population density of *T. poliocephalus* in Fusui Nature Reserve (6 langurs/km^2^) was reported to be significantly higher than that in Nonggang Nature Reserve (1.4 langurs/km^2^), while the home range of langurs in Nonggang (69.3 ha) was significantly larger than that in Fusui (19 ha) (Zhou 2005). The population density of François’ langurs in the Kuankuoshui Nature Reserve was about 4 langurs/km^2^ observed in this study. The grid method home range was 50.7 ha. The population density and home range of François’ langurs in Kuankuoshui were between those of Nonggang and Fusui Nature Reserve (Table 1).

The home range size has also been reported to be related to the group size (Isbell 1991; Ostro et al. 1999). A significant positive correlation was found between home range and daily travel distance and foraging group size of a mountain gorilla group (*Gorilla gorilla beringei*), which was related to the increase in food demand caused by the group's size increase (Clutton-Brock and Harvey 1977). The target monkey group consisted of 12 monkeys, including eight adult individuals, and the home range was 50.7 ha. There were also 12 monkeys in Nonggang area, including nine adults, with a home range of 69.3 ha (Table 1) (Zhou 2005); there were seven monkeys in Fusui area, including four adults, with a home range of only 19 ha (Huang et al. 2011). A positive relationship was also found in *Rhinopithecus roxellanae* in Shennongjia Nature Reserve, where a population of 236 individuals had a home range of 2250 ha, while a small population of 62 individuals had a home range of only 1240 ha (Fan et al. 2019). There were several hundred *R. bieti* living in high-altitude forest areas, with a home range of 2525 ha (Kirkpatrick 1996). In general, the larger the primate population, the larger of the corresponding home range.

### Influence of altitude on the target group size and the daily travel distance of François’ langurs

The higher altitude may be associated with larger groups. The altitude of Kuankuoshui Nature Reserve is between 650 and 1750 m, and the average unit group size of François’ langurs included 6.73 individuals (Yao et al. 2016), while the altitude of Guangxi Nonggang Nature Reserve ranges between 300 and 700 m, and the average unit group size of François’ langurs was 5.85 individuals (Tang et al. 2011). The François' langurs can form a larger group of 8–14 individuals at an altitude of more than 1300 m, and form a small group of 5–7 individuals at an altitude of 700–1000 m in Yezhong Nature Reserve (Deng and Zhou 2018). In the presently studied area (altitude of 800–1400 m), the target monkey group had a large unit of 12 monkeys, and there were nine monkeys in another group that were distributed in the same area as the unit monkey group. The formation of this group size might be related to the influence of altitude on temperature and food resources. The favorite food species of François’ langurs are the plants such as Rosaceae, grapes, Actinidiaceae (Yu et al. 2004), but there were mainly coniferous broad-leaved mixed forests in the high-altitude area of Kuankuoshui Nature Reserve, which had few edible food resource species. Nonggang Nature Reserve in Guangxi has a low altitude and latitude; the vegetation is a mountainous evergreen seasonal rainforest type that is rich in food resources. A similar pattern could be found in the snub-nosed monkeys. *R. brelichi* live at an altitude of 1400–2100 m, usually in small groups of 22–56 monkeys (Guo et al. 2020). *R. roxellanae* in Shennongjia lives at an altitude of 1000–3100 m, usually in the group sizes of 70–210 monkeys (Cao et al. 2019). *R. bieti* in Xiangguqing of Baima Snow Mountain Reserve, Yunnan, live at an altitude of 3600–4100 m, forming a super-large group of 480 monkeys, which was separated by manual intervention into a small group and one large group of 360 individuals (Ren et al. 2016). Therefore, in order to cope with the high-altitude habitat, low temperature, and lack of food resources, the two groups of colobus monkeys studied formed a larger group structure and large home range. A large home range may improve the diversity of food to extend the repeated use of the same feeding point cycle, which is a good strategy to adapt to high-altitude habitats.

The altitude difference in the habitat of François’ langurs may affect their daily travel distance. The daily travel distance of François’ langurs in the summer-autumn season (517 m) was significantly smaller than that in the winter-spring season (785 m), which might be related to the reduced food resources in the winter-spring season, the lower availability of young leaves, flowers, and fruits, and the increased time spent by monkeys looking for food. The daily travel distance in this study was smaller than that of François’ langurs living in the low-altitude areas of Nonggang in the rainy season (710 m) and in the dry season (836 m) (Huang et al. 2011). In January, the shortest daily travel distance of François’ langurs was observed in Kuankuoshui Reserve, which might be due to the cold climate and heavy fog, where François’ langurs minimized activity to reduce the need for energy consumption.

### Home-range size and research methods

The different analysis methods may lead to different results when calculating the area of an animal’s home range (Macdonald et al. 1980). We used two kinds of analysis methods that are most commonly used: the grid cell method and the MCP method (White and Garrott 1990). The grid method can reflect the actual utilization of home range by the target animals; however, the calculated home range is usually smaller than the actual area (Sterling et al. 2000). The MCP method estimates the home range by calculating the polygon area enclosed by the edge of the activity area of the research subjects (Harris et al. 1990; White and Garrott 1990). The area calculated by this method tends to be larger, as there are places within the polygon that the target animal might not use (Ostro et al. 1999; Powell 2000; Burgman and Fox 2003). In this study, the home range of François’ langurs calculated by the grid method was 50.7 ha, and that calculated by the MCP method was 150.6 ha, which was significantly larger than the former. The home range of François’ langurs calculated by the MCP method in summer, autumn, winter, and spring was 20.8, 66.3, 72.7, and 76.7 ha, respectively. Except for the summer season, each season's MCP home range was similar in size to the home range calculated by the grid method. Therefore, the grid method might better reflect the actual home range or core home range of François’ langurs, while the MCP method could reflect the potential habitat range that François’ langurs may use.

### Home range use and groups conflict

The home range of most primates overlaps and reveals obvious territorial behaviors (Clutton-Brock and Harvey 1977; Bennett 1983; Ostro et al. 1999). In this study, there is home range overlapping of François’ langurs in different seasons from 21.6 to 27.7% and from 11.7 to 37.9% in different months. This indicated that François’ langurs can utilize the same area in different months or seasons. The home range of overlap between different groups is low. Adjacent groups rarely go in to the home range of other groups. We only observed our target group encounter another group once during the study period. At this time, they vocally indicated their home range at a distance, without intense conflict. A similar situation was also observed in the Daheba protection station of Mayanghe National Nature Reserve when we conducted a François’ langurs behavior study in 2016–2017. The results showed that François’ langurs of different group units engaged in home-range overlap but did not show strong territorial behavior and generally did not simultaneously use the overlapping home-range portion.

## Conclusions

Compared with the François’ langurs living in low-altitude areas, François’ langurs living in high-altitude areas generally formed larger groups with larger home ranges and smaller daily travel distances. Therefore, it was concluded that the colobines in high-altitude areas could well adapt to the environment of high altitude and low temperature by adopting the habitat adaptation strategy with large home range and short daily travel distance.

## Data Availability

There is no data was used for this article.

## Appendix 1

(See Table

**Table 2:** The number of sampling days, observation time, and location recording

Month	Effective days	Effective time (*h*)	Full days	Site record
Apr. 2008	8	26	2	52
May. 2008	7	30	3	60
Jun. 2008	10	31	4	62
Jul. 2008	9	28	3	56
Aug. 2008	8	30	3	60
Sept. 2008	6	26	2	52
Oct. 2008	9	48	4	96
Nov. 2008	12	75	6	150
Dec. 2008	14	72	5	144
Jan. 2009	5	10	1	20
Feb. 2009	9	35	2	70
Mar. 2009	7	48	4	96
Total	102	459	39	918

Effective days = the number of days is observed target group activities; Full days = the number of unique days on which the target group was observed from morning to evening

2).

## Appendix 2

(See Table

**Table 3:** Availability of different parts of the plant in different months based on the data of 20 quadrates at Kuankuoshui Nature Reserve

Month	Young Leaves℅	Flower℅	Fruit℅
Apr. 2008	24.8	19.4	2.6
May. 2008	24.8	28.1	6.3
Jun. 2008	21.8	2.6	15.6
Jul. 2008	13.9	2.0	9.5
Aug. 2008	7.1	7.3	9.0
Sept. 2008	3.4	8.0	9.5
Oct. 2008	1.4	6.7	10.0
Nov. 2008	0.8	5.2	7.9
Dec. 2008	0.7	5.2	6.9
Jan. 2009	0.0	6.0	5.8
Feb. 2009	0.0	4.0	6.3
Mar. 2009	0.7	3.3	2.1

^3^).
